# Elevated serum 1,25(OH)_2_-vitamin D_3_ level attenuates renal tubulointerstitial fibrosis induced by unilateral ureteral obstruction in *kl/kl* mice

**DOI:** 10.1038/srep06563

**Published:** 2014-10-09

**Authors:** Yujing Sun, Gengyin Zhou, Ting Gui, Aiko Shimokado, Masako Nakanishi, Kosuke Oikawa, Fuyuki Sato, Yasuteru Muragaki

**Affiliations:** 1 First Department of Pathology, Wakayama Medical University School of Medicine, 811-1 Kimiidera, Wakayama 641-0012, Japan; 2 Department of Pathology, School of Medicine, Shandong University, Jinan Wen Hua Xi Road 44, Jinan 250012, PR China; 3 Current address: Department of Nephrology and Hypertension, Inselspital, Bern 3010, Switzerland.

## Abstract

Previous studies have suggested that Klotho provides reno-protection against unilateral ureteral obstruction (UUO)-induced renal tubulointerstitial fibrosis (RTF). Because the existing studies are mainly performed using heterozygous Klotho mutant (HT) mice, we focused on the effect of UUO on homozygous Klotho mutant (*kl/kl*) mice. UUO kidneys from HT mice showed a significantly higher level of RTF and TGF-β/Smad3 signaling than wild-type (WT) mice, whereas both were greatly suppressed in *kl/kl* mice. Primary proximal tubular epithelial culture cells isolated from *kl/kl* mice showed no suppression in TGF-β1-induced epithelial mesenchymal transition (EMT) compared to those from HT mice. In the renal epithelial cell line NRK52E, a large amount of inorganic phosphate (Pi), FGF23, or calcitriol was added to the medium to mimic the *in vivo* homeostasis of *kl/kl* mice. Neither Pi nor FGF23 antagonized TGF-β1-induced EMT. In contrast, calcitriol ameliorated TGF-β1-induced EMT in a dose dependent manner. A vitamin D_3_-deficient diet normalized the serum 1,25 (OH)_2_ vitamin D_3_ level in *kl/kl* mice and enhanced UUO-induced RTF and TGF-β/Smad3 signaling. In conclusion, the alleviation of UUO-induced RTF in *kl/kl* mice was due to the TGF-β1 signaling suppression caused by an elevated serum 1, 25(OH)_2_ vitamin D_3_.

Klotho was first identified in close association with a premature onset of aging phenotype in mice. Homozygous Klotho mutant mice (*kl/kl*) exhibit a shortened life span, infertility, ectopic calcification, skin atrophy, pulmonary emphysema, and osteoporosis, which resemble an aged state in humans^1^. Mouse Klotho is predominantly expressed in the kidneys and slightly in the brain^2^. Two forms of Klotho have been identified: membrane Klotho and secreted Klotho. Membrane Klotho functions as a co-receptor of the FGF23 receptor^3^,^4^. FGF23, a member of the fibroblast growth factor (FGF) family, is a bone-derived hormone that exerts its function in the kidneys, to help maintain homeostasis in phosphate and vitamin D metabolism by regulating the sodium phosphate co-transporter and the key enzymes for vitamin D metabolism CYP27B1 and CYP24A1^5^,^6^,^7^,^8^. Similar to FGF23-deficient mice, *kl/kl* mice also exhibit hyperphosphatemia and an increased serum 1,25 (OH)_2_ vitamin D_3_ level^1^, which are suspected to be the primary cause of premature aging because restricting vitamin D intake improves the *kl/kl* phenotype^8^,^9^. Secreted Klotho is formed via either alternative splicing of the gene or shedding of the extracellular domain into the extracellular space and subsequently into the circulation^2^,^10^.

Renal tubulointerstitial fibrosis (RTF) is the final common pathological condition of chronic kidney disease (CKD) regardless of the underlying cause^11^. RTF is characterized by an excess accumulation of extracellular matrix (ECM), fibroblast activation, and a loss of functioning nephrons^12^. Recent studies have suggested that Klotho plays an important role in RTF. Kidney injury, such as ischemia-reperfusion, peritoneal cisplatin injection, or angiotensin II administration, induce Klotho deficiency, whereas exogenous Klotho protein attenuates kidney injury^13^,^14^,^15^,^16^,^17^. In a mouse model of CKD, unilateral ureteral obstruction (UUO)-induced RTF is exaggerated in heterozygous Klotho mutant mice (HT) and is ameliorated in Klotho over-expressing mice. Secreted Klotho has been proven to interfere with TGF-β^18^ and Wnt/β-catenin^19^ signaling to suppress UUO-induced RTF.

*Kl/kl* mice exhibit phenotypes that are very different from wild-type or HT mice. Hyperphosphatemia and high 1, 25(OH)_2_ vitamin D_3_ and/or FGF23 are the most noticeable serologic features. ECM deposition is increased in *kl/kl* mice even without UUO and is thought to be due to Klotho deficiency^20^. However, an activated form of vitamin D and its analogues have been shown to protect the kidneys from fibrosis due to various kidney injuries^21^,^22^. Also, 1, 25(OH)_2_ vitamin D_3_ reduces gene expression related to TGF-β-induced fibrosis in human uterine leiomyoma cells^23^. An active form of the vitamin D_3_ analogue maxacalcitol recruits a PPM1A/VDR complex to phosphorylated Smad3 to accelerate its dephosphorylation^24^. Thus, the outcome of UUO-induced RTF becomes uncertain because of the opposing effect of an elevated vitamin D and Klotho deficiency. Because all of the existing studies on the effect of Klotho on UUO-induced RTF were performed using HT mice, whether the disrupted homeostasis in *kl/kl* mice has an effect is not known.

In this study, we compared the degree of UUO-induced RTF among wild-type, HT, and *kl/kl* mice and analyzed the underlying mechanisms.

## Results

### UUO-induced RTF is the mildest in *kl/kl* mice

**Figure 1:**
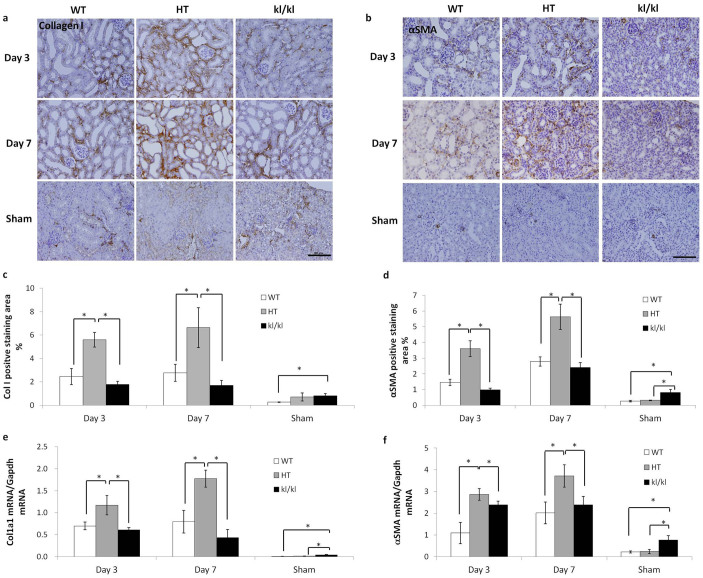
*Kl/kl* kidneys showed the mildest UUO-induced renal tubulointerstitial fibrosis. (a, b): Representative pictures for immunohistochemistry of kidney sections from WT, HT, and *kl/kl* mice for collagen I (a) and αSMA (b). Scale bar: 100 μm (c, d) Quantification of positive staining areas for collagen I (c) and αSMA (d). (e, f) Real-time PCR for Col1a1 (e) and αSMA (f). Note that UUO-induced collagen I deposition and fibroblast activation were the most severe in HT mice at the protein and mRNA levels and were largely abolished in *kl/kl* mice. Data are presented as the means ± S.D. *P < 0.05 (n = 5).

Six week-old wild-type (WT), heterozygous Klotho mutant (HT), and *kl/kl* mice were subjected to UUO for 3 days or 1 week. The expression of RTF markers collagen I and αSMA was assessed by immunohistochemistry and quantitative real-time PCR. RTF developed after the UUO operation in a time dependent manner in WT and HT mice compared to the sham-operated control groups. The expression of RTF markers was much higher in HT mice than WT mice, which is corroborated by previous reports^18^,^20^. In the sham-operated groups, the expression of RTF markers was comparable between the WT and HT mice, but was increased more than 2-fold in the *kl/kl* mice. Conversely, *kl/kl* mice showed only a slight increase in the expression of fibrosis markers after UUO (Fig. 1).

### TGF-β signaling is suppressed in *kl/kl* mice

**Figure 2:**
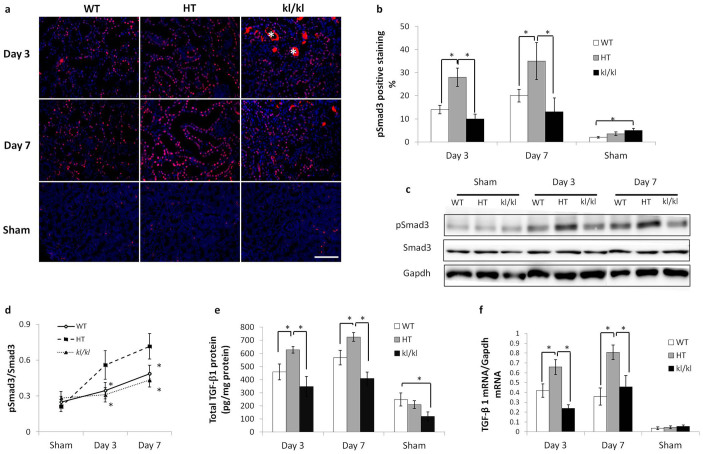
UUO-induced TGF-β1/Smad3 signaling is ameliorated in *kl/kl* mice. (a) Immunofluorescence for phosphorylated (p)-Smad3. Scale bar: 100 μm (b) Comparison of the number of pSmad3-positive nuclei among WT, HT, and *kl/kl* kidneys. (c) Western blot for pSmad3 in UUO-treated kidneys. Full-length blots are included in the supplementary information. (d) Comparison by quantification of pSmad3 in western blot among WT, HT, and *kl/kl* kidneys. (e) The amount of TGF-β1 protein in WT, HT, and kl/kl kidneys as measured by ELISA. (f) TGF-β1 mRNA level as measured by real-time PCR. Note that UUO-induced TGF-β signaling was enhanced in HT kidneys compared to WT kidneys and was ameliorated in *kl/kl* kidneys. Data are presented as the means ± SD. *P < 0.05 (n = 5).

Previous studies showed that Klotho protein interferes with TGF-β1 binding to its receptor, resulting in suppressed TGF-β signaling^18^. As a result, UUO-induced RTF is exaggerated in HT mice^20^. To examine whether the suppressed expression of RTF markers in *kl/kl* mice is a result of inhibited TGF-β signaling, the phosphorylation of Smad3 and TGF-β1 expression was assayed. In parallel with RTF markers, after UUO, phosphorylated Smad3 (pSmad3) and TGF-β1 expression were the highest in HT mice and were significantly suppressed in *kl/kl* mice. Interestingly, in the sham-operated groups, the *kl/kl* mice had a higher percentage of pSmad3 positive staining cells than the other two groups, whereas this group had the lowest TGF-β1 content, suggesting that Smad3 phosphorylation in the sham-operated *kl/kl* mice was not a result of increased TGF-β1 (Fig. 2).

### No suppression of the TGF-β1-induced epithelial-mesenchymal transition is observed in primary cultured proximal tubular epithelial culture cells isolated from *kl/kl* mice

**Figure 3:**
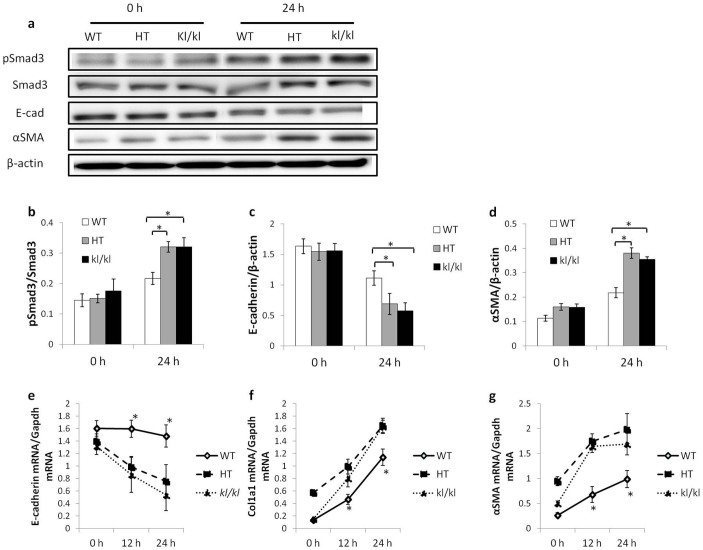
TGF-β1-induced EMT is enhanced in proximal tubular cells isolated from HT and *kl/kl* kidneys compared to WT kidneys. (a) Representative western blots for pSmad3, Smad3, E-cadherin (E-cad), αSMA, and β-actin. Full-length blots are included in the supplementary information. (b–d) Comparison by quantification of western blots for pSmad3 (b), E-cadherin (c), and αSMA (d). (e–g) Real-time PCR for E-cadherin (e), Col1a1 (f), and αSMA (g). Note that unlike in vivo, no difference in EMT markers was detected between HT and *kl/kl* cultured proximal tubular cells. Data are presented as the means ± SD. *P < 0.05 compared to the HT group at the same time point. Experiments were repeated in triplicate.

Because *kl/kl* mice have disrupted phosphate and calcium (Pi/Ca) homeostasis, determining whether the suppressed TGF-β/Smad3 signaling is a result of complete depletion of Klotho or disrupted Pi/Ca homeostasis, most noticeably high phosphate, high FGF23, and high 1, 25(OH)_2_ vitamin D_3_, is difficult. To address the underlying molecular mechanism, we compared the expression of molecules involved in epithelial-mesenchymal transition (EMT) using primary cultured proximal tubular epithelial cells (PTECs) from WT, HT, and *kl/kl* mice. TGF-β1-induced EMT was enhanced in HT and *kl/kl* cells compared to WT cells. The EMT markers showed higher αSMA and lower E-cadherin mRNA and protein expression at the indicated time point (Fig. 3). Smad3 phosphorylation was also enhanced in HT and *kl/kl* cells. Unlike UUO kidneys, *kl/kl* cells showed no suppression of TGF-β1-induced EMT and Smad3 phosphorylation compared to HT cells. These results suggest that the suppressed TGF-β/Smad3 signaling observed in UUO-induced RTF in *kl/kl* kidneys was a result of disrupted Pi/Ca homeostasis and not complete Klotho depletion (Fig. 3).

### TGF-β1-induced EMT is suppressed not by high phosphate or FGF23 but by 1,25(OH)_2_ vitamin D_3_ in a dose-dependent manner

Previous studies showed that in *kl/kl* mice, serum phosphate and 1,25(OH)_2_ vitamin D_3_ increase sharply^1^ and serum FGF23 concentration increased 2,000-fold compared to WT mice^4^. To identify an individual factor(s) that contribute to suppressed UUO-induced RTF expression in *kl/kl* mice, we used the normal kidney epithelial cell line NRK52E, and these cells were cultured in the presence of high phosphate (2 or 4 mM), FGF23 (0.2 or 2 μg/ml), or 1,25(OH)_2_ vitamin D_3_ (0.1 or 1 μg/ml) to evaluate their effect on TGF-β1-induced EMT. In the cells treated with TGF-β1 together with phosphate or FGF23 for 12 or 24 h, TGF-β1-induced EMT was not affected at the mRNA or protein level compared to cells treated with TGF-β1 alone. In contrast, 1,25(OH)_2_ vitamin D_3_ ameliorated TGF-β1-induced Smad3 phosphorylation, αSMA expression, and E-cadherin suppression in a dose dependent manner at the mRNA and protein levels. This suppressive effect of 1,25(OH)_2_ vitamin D_3_ on TGF-β-induced EMT lasted for 72 h after TGF-β treatment (supplementary Fig. 1).

**Figure 4:**
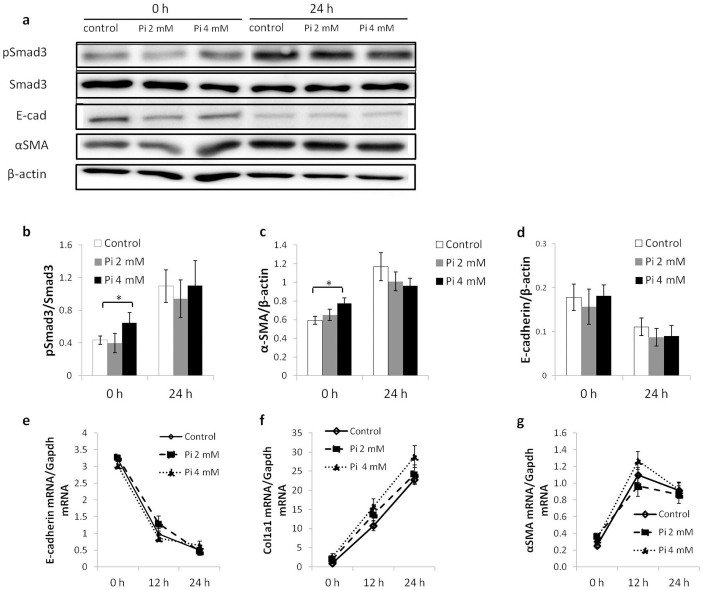
TGF-β1-induced EMT in NRK52E cells is not influenced by a high concentration of phosphate. (a) Representative western blots for pSmad3, Smad3, E-cadherin (E-cad), αSMA, and β-actin. Full-length blots are included in the supplementary information. (b–d) Comparison by quantification of western blots for pSmad3 (b), E-cadherin (c) and αSMA (d). (e-g) Real-time PCR for E-cadherin (e), Col1a1 (f), and αSMA (g). A high concentration of phosphate does not enhance TGF-β1-induced EMT. Note that basal Smad3 phosphorylation and αSMA protein expression are enhanced in the presence of a high phosphate level. Data are presented as the means ± SD. *P < 0.05. Experiments were repeated in triplicate.

**Figure 5:**
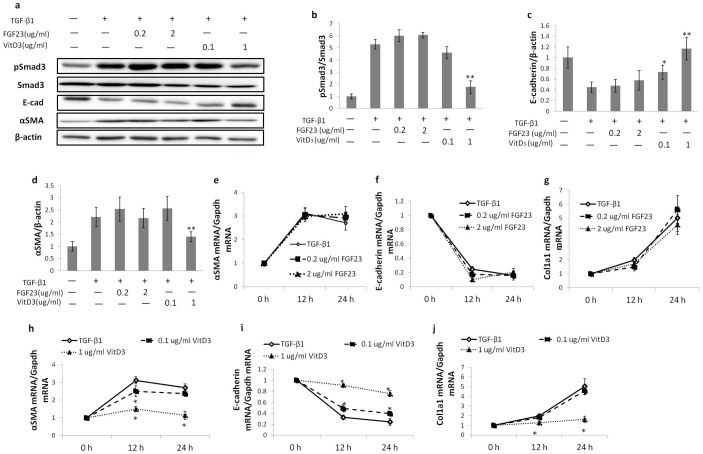
TGF-β1-induced EMT in NRK52E cells is suppressed by calcitriol. (a) Representative western blots for pSmad3, Smad3, E-cadherin, αSMA, and β-actin. Full-length blots are included in the supplementary information. (b–d) Comparison by quantification of western blots for pSmad3 (b), E-cadherin (c), and αSMA (d). (e–j) Real-time PCR results for E-cadherin (e, h), Col1a1 (f, i), and αSMA (g, j) in the presence of either FGF23 (e, f, g) or calcitriol (h, i, j). FGF23 have no effect on TGF-β1-induced EMT at neither low nor high concentration. Note that calcitriol ameliorates TGF-β1 induced EMT in a dose-dependent manner. Data are presented as the means ± SD. *P < 0.05 versus cells treated with TGF-β1 only. Experiments were repeated in triplicate.

Interestingly, the cells cultured with high phosphate (4 mM) for 48 h had more Smad3 phosphorylation and higher αSMA expression compared to cells cultured in normal DMEM, although TGF-β1 was absent. These results may explain the increased fibrosis markers observed in sham-operated *kl/kl* mice (Figs. 4 and 5).

### Vitamin D-free diet normalizes serum 1,25(OH)_2_ vitamin D_3_ level and enhances the expression of UUO-induced RTF markers in *kl/kl* mice

**Figure 6:**
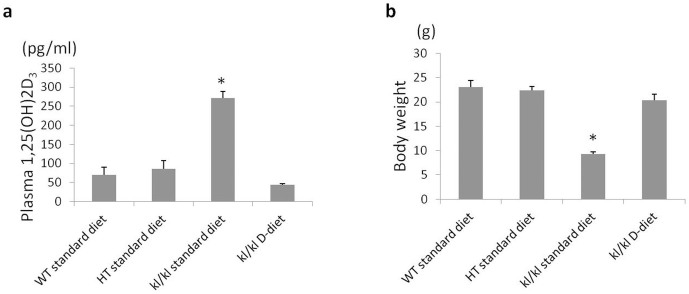
A 1,25(OH)_2_-vitamin D_3_-deficient diet (D-diet) normalizes serum active vitamin D levels in *kl/kl* mice and rescues the *kl/kl* phenotype. (a) Serum level of active vitamin D. (b) Body weight. Data are presented as the means ± SD. *P < 0.05 vs. WT (n = 4).

A vitamin D-free diet (D-diet) has been reported to maintain phosphate homeostasis and improve the *kl/kl* phenotype^8^,^9^. In this study, we fed *kl/kl* mice on a D-diet to determine whether the decreased vitamin D level could enhance UUO-induced RTF. In parallel with previous studies, the D-diet normalized serum 1,25(OH)_2_ vitamin D_3_ and increased the body weight of the kl/kl mice, such that they were indistinguishable from their WT littermates (Fig. 6).

**Figure 7:**
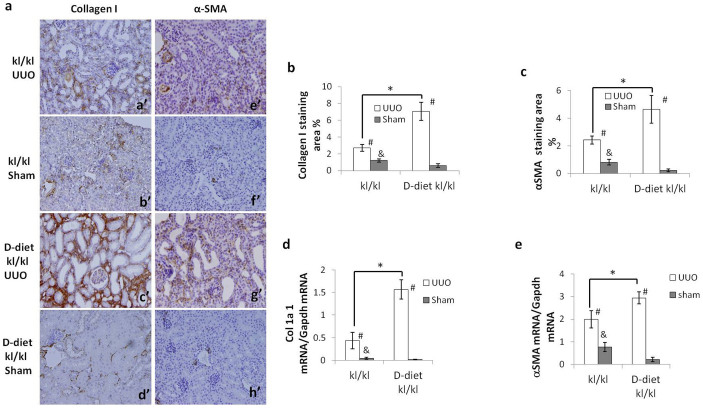
A vitamin D3-free diet enhances UUO-induced RTF in *kl/kl* mice to the same level as HT mice. (a) Representative immunohistochemistry of kidney sections for collagen I (a–d) and αSMA (e–h) (b, c) Comparison by quantifying the positive staining area for collagen I (b) and αSMA (c). (d, e) Real-time PCR for Col1a1 (d) and αSMA (e). A vitamin D3 free diet significantly enhances UUO-induced protein and mRNA expression of collagen I and αSMA in *kl/kl* mice. Data are presented as the means ± SD. *P < 0.05, #p < 0.05 vs. sham-treated kidneys, &p < 0.05 vs. D-diet *kl/kl* sham-treated kidneys (n = 5).

After the UUO operation, based on immunohistochemistry, expression of collagen I and αSMA was enhanced by the D-diet, with 3- and 4-fold increases, respectively, compared to *kl/kl* mice fed a standard diet, and mRNA expression showed a similar trend (Fig. 7).

**Figure 8:**
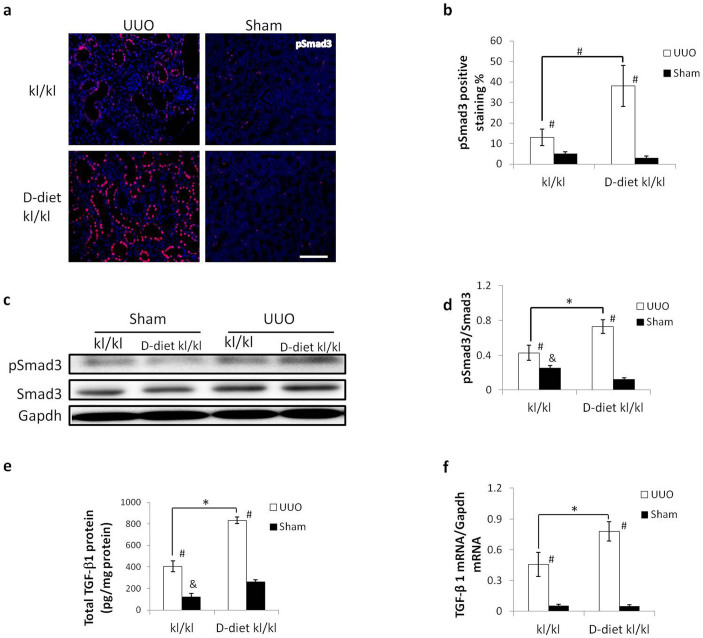
TGF-β1 signaling in *kl/kl* mice is enhanced by a vitamin D3 free diet. (a) Representative immunofluorescence for phosphorylated (p)-Smad3. (b) Western blot for pSmad3 in UUO- or sham-treated kidneys. Full-length blots are included in the supplementary information. (c) Comparison of the number of pSmad3-positive nuclei. (d) Comparison of pSmad3 to total Smad3 ratio in western blots. (e) TGF-β1 protein expression in the kidneys as measured by ELISA. (f) TGF-β1 mRNA expression as measured by real-time PCR. Amelioration of UUO-induced activation of TGF-β1 signaling in *kl/kl* mice was abolished by a vitamin D3 free diet. pSmad3 positive nuclei and TGF-β1 expression were increased at the protein and mRNA levels. Data are presented as the means ± SD. *P < 0.05 (n = 5), #p < 0.05 vs. sham treated kidneys. &P < 0.05 vs. D-diet *kl/kl* mice.

In addition, the D-diet enhanced TGF-β/Smad3 signaling in UUO-induced RTF in *kl/kl* mice. The percentage of pSmad3-positive cells increased about 2.5-fold as a result of vitamin D withdrawal. ELISA was performed to measure the TGF-β1 content in kidney lysates. Protein and mRNA expression of UUO-induced TGF-β1 increased about 2-fold using the D-diet. Interestingly, in the sham-operated *kl/kl* mice fed the standard diet, TGF-β1 protein expression was lower than the *kl/kl* mice fed the D-diet, while there were more pSmad3 positive cells in *kl/kl* mice fed the standard diet (Fig. 8).

Note that in sham-operated *kl/kl* mice fed a standard diet, the expression of RTF markers and activation of TGF-β/Smad3 signaling are slightly but significantly higher than *kl/kl* mice fed a D-diet. The results suggest that increased RTF marker expression in sham-operated *kl/kl* mice was not a result of Klotho depletion but the disrupted Pi/Ca homeostasis (Figs. 7 and 8).

## Discussion

In this study, we demonstrated that UUO-induced RTF was greatly suppressed in *kl/kl* mice compared to HT mice. Conversely, slightly but significantly more severe renal fibrosis was observed in sham-operated *kl/kl* kidneys than WT and HT kidneys. Because *kl/kl* mice exhibit a variety of abnormalities, among which phosphate retention and high serum 1, 25(OH)_2_ vitamin D_3_ are the most prominent changes, we hypothesized that disrupted Pi/Ca homeostasis in *kl/kl* mice may play an important role in the mild renal fibrosis in sham-operated mice and the suppression of RTF in UUO-operated mice.

We observed that HT mice exhibit more severe UUO-induced RTF compared to the WT group, which is consistent with previous reports^20^. Klotho has been proven to protect the kidneys from injury-induced fibrosis by counteracting TGF-β/Smad3 and Wnt signaling^18^,^19^. Klotho deficiency renders the kidneys more susceptible to acute insults, while exogenous Klotho expression attenuates renal fibrosis due to various causes^13^,^14^,^18^,^20^,^25^. In rodent models, renal damage caused by several different kinds of stress, such as acute inflammatory stress and sustained circulatory stress, could suppress Klotho expression^26^,^27^. In the human kidneys, decreased Klotho expression occurs as early as CKD stage 2^28^. However, unexpectedly, UUO-induced RTF in *kl/kl* mice was greatly attenuated. Because TGF-β/Smad3 signaling is believed to be the main driving force toward post-UUO RTF^29^,we examined TGF-β/Smad3 signaling, which turned out to be significantly suppressed in *kl/kl* kidneys. To identify the cause of this suppression, we examined TGF-β-induced EMT using primary cultured PTECs to exclude the humoral effects. Interestingly, unlike *in vivo* experiments, TGF-β1-induced EMT was not suppressed in cultured *kl/kl* PTECs, and there was an increase in Smad3 phosphorylation. These results suggest that the suppression of UUO-induced RTF in *kl/kl* mice might result from a disturbance in Pi/Ca homeostasis due to deficiency of Klotho. We have not investigated ERK/MAPK activation in cultured *kl/kl* PTECs because the increase in Smad3 phosphorylation was significant, but it might be important to examine non-canonical TGF-β signaling, which also plays an important role in EMT.

Next, because phosphate retention, high serum FGF23, and high serum 1, 25(OH)_2_ vitamin D_3_ are the most prominent changes in *kl/kl* mice^30^, we examined these factors individually using in vitro cultures. NRK52E cells were challenged with TGF-β1 in the presence of high phosphate, FGF23, or calcitriol (1, 25(OH)_2_ vitamin D_3_). TGF-β1-induced EMT was not affected by the presence of high phosphate or FGF23, but was inhibited by calcitriol in a dose dependent manner. These results suggest that a high level of 1, 25(OH)_2_ vitamin D_3_ but not phosphate or FGF23 may be the cause of the suppressed UUO-induced RTF in *kl/kl* mice.

To confirm this mechanism, we sought to eliminate the effect of vitamin D in *kl/kl* mice by feeding a D-diet and determining the degree of UUO-induced RTF. The D-diet normalized 1, 25(OH)_2_ vitamin D_3_ and phosphate levels in the blood of *kl/kl* mice and completely rescued the *kl/kl* phenotype, which is in agreement with previous reports^8^. As we predicted, the D-diet enhanced UUO-induced RTF in *kl/kl* mice to the same levels as HT mice. In addition, TGF-β signaling was enhanced by eliminating vitamin D. Paricalcitol, a synthetic vitamin D analogue, has been reported to attenuate renal interstitial fibrosis in obstructive nephropathy via repressing expression of TGF-β1 and its type I receptor^31^. Moreover, vitamin D analogs are reported to ameliorate proteinuria and kidney injury by blocking Wnt/β-catenin signaling^32^. We therefore conclude that an elevated level of 1, 25(OH)_2_ vitamin D_3_ in *kl/kl* mice suppresses TGF-β/Smad3 signaling, resulting in amelioration of UUO-induced RTF.

In sham-operated mice, there was no significant difference in the expression of RTF markers between WT and HT kidneys, whereas expression was significantly albeit mildly increased in *kl/kl* kidneys compared to WT and HT kidneys. Sugiura, H et al^20^ suggested that this result may be due to failed TGF-β/Smad3 inhibition related to the absence of Klotho. However, our results did not support this hypothesis because the *kl/kl* PTECs expressed the same levels of EMT markers as WT and HT PTECs treated with TGF-β1. In addition, *kl/kl* mice fed a D-diet did not have any RTF onset in the sham-operated group. Interestingly, NRK52E cells cultured for 48 h in the medium containing a high concentration of phosphate (4 mM) showed Smad3 phosphorylation and increased αSMA expression compared to the control medium (Pi 1 mM), suggesting that an increased expression of RTF markers in sham-operated *kl/kl* mice may be a result of a disrupted Pi/Ca homeostasis, that is, a high concentration of phosphate and not a result of failed TGF-β/Smad3 inhibition by Klotho. However, at this time, we cannot explain how a high concentration of phosphate phosphorylates Smad3.

In conclusion, we are the first to report that UUO-induced TGF-β/Smad3 signaling is suppressed in *kl/kl* mice by an increased level of 1, 25(OH)_2_ vitamin D_3_, resulting in amelioration of UUO-induced RTF. We identified 1, 25(OH)_2_ vitamin D_3_ to be a powerful suppressor of TGF-β/Smad3 signaling and protect the kidneys from RTF.

## Methods

### Animals, diets, UUO animal models, and tissue preparation

All experimental protocols were approved by the Animal Studies Committee of Wakayama Medical University. The methods were carried out in accordance with the approved guideline. This study used *klotho*/Jcl mice, which were purchased from CLEA Japan and described elsewhere^1^. Mice were raised in a standard facility with free access to food and water.

Animals were fed a standard diet or vitamin D_3_ free diet (D-diet). In the D-diet groups, heterozygous *klotho* female mice were maintained on a vitamin D_3_ free diet (PMI nutrition international, Inc.) after pregnancy, in which the composition was identical to the standard diet except for missing vitamin D_3_. The offspring of D-diet heterozygous *klotho* females were also fed a D-diet throughout the experiments.

Six- to eight-week-old male wild-type (WT), heterozygous (HT), and *kl/kl* mice from the standard diet and D-diet groups were used to perform UUO. UUO surgery was performed as described elsewhere^33^,^34^. In short, male mice from each group (n = 5) were anesthetized by an intraperitoneal injection of ketamine (100 mg/kg body weight) and xylazine (5 mg/kg body weight). A left-flank incision was made to expose the left ureter, and then ligation was done with a 5-0 silk suture. The sham groups (n = 3) received the same procedure except for ureteral ligation.

At the time of sacrifice, the ligated kidneys (or sham kidneys) were removed and cut transversely. Tissues were fixed with 4% paraformaldehyde for histopathological examination or lysed by denaturing lysis buffer (50 mM Tris-HCl, pH 7.4, 1% triton X, 150 mM NaCl, 1 mM EDTA) with addition of a phosphatase inhibitor (2.5 mM sodium orthovanadate, 10 mM NaF, and 10 mM β-glycerol phosphate) and proteinase inhibitor (Roche). Lysed samples were stored in −80°C until use for western blot analysis or ELISA.

### Immunohistochemistry and immunofluorescent staining

Immunohistochemistry was performed on paraffin sections using a microwave-based antigen retrieval technique. The antibodies used in this study include collagen I (Southern Biotechnology, Birmingham, AL), pSmad3 (Cell Signaling, Danvers, MA), αSMA (Sigma) and Fsp1 (Abcam). After incubation with the primary antibodies, sections were treated with a Vectastain ABC kit (Vector Laboratories, Birlingame, CA) according to the manufacturer's instructions. For immunofluorescent staining, a Cy3-anti-rabbit IgG (Sigma Aldrich) was used as a secondary antibody.

### Quantitative real-time polymerase chain reaction (qRT-PCR)

SYBR Green PCR Master Mix (Takara Bio, Inc., Japan) was used to analyze mRNAs. Each sample was measured in duplicate, and gene expression levels were normalized to glyceraldehyde 3-phosphate dehydrogenase (Gapdh). Sequences of the primers used for qRT-PCR are as follows: mouse-Col1a1 forward 5′-CAA CCT GGA CGC CAT CAA G-3′ and reverse 5′-CAG ACG GCT GAG TAG GGA ACA-3′; rat-Col1a1 forward 5′-AGT GCA TTC AAC CTT ACC AA-3′ and reverse 5′-TCA AGC AAG AGG ACC AAG C-3′; mouse and rat-Fsp1 forward 5′-GAA GTG AAG ACT CCT CAG ATG-3′ and reverse 5′-CAC TAT GCT CAC AGC CAA C-3′; mouse-TGF-β1 forward 5′-AAC TAT TGC TTC AGC TCC AGA GAG A-3′ and reverse 5′-AGT TGG ATG GTA GCC CTT G-3′; rat-TGF-β1 forward 5′-TGG AGC AAC ACG TAG AAC TCT-3′ and reverse 5′-AGT TGG ATG GTA GCC CTT G-3′; mouse-αSMA forward 5′-AGA TCA CAG CCC TCG CA-3′ and reverse 5′-AGA GTA CTT GCG TTC TGG AG-3′; rat-αSMA forward 5′-AGA TCA CAG CCC TCG CT-3′ and reverse 5′-AGA ATA TTT GCG TTC TGG AG-3′; rat-αSMA forward 5′- ATG ATG AAG AGG GAG GTG G-3′ and reverse 5′- CAT CAT TTC GAA TCA CTT CC-3′; mouse-Gapdh forward 5′-CCC ATG TTT GTG ATG GGT GT-3′ and reverse 5′-GTG ATG GCA TGG ACT GTG GT-3′^35^.

### Western Blot Analysis

Tissue or cell lysate (20 μg) was separated by SDS-PAGE and blotted on polyvinylidene difluoride membranes (Millipore, Billerica, MA). The primary antibodies were rabbit anti-pSmad3 (Acris Antibodies, Herford, Germany), rat anti-E-cadherin (Sigma Aldrich), mouse anti-αSMA (Sigma Aldrich), rabbit anti-Smad3 (Abcam), mouse anti-β-actin (Sigma Aldrich), and goat anti-GAPDH(Santa Cruz). The signals were developed with the ECL Plus detection system (Amersham Bioscience, Buckinghamshire, United Kingdom).

### TGF-β1 ELISA assay

Kidney cortices were dissected and homogenized in denaturing lysis buffer containing 100 mM NaCl, 1% Triton X-100, and 0.05 M Tris-HCl, pH7.4. Protein concentrations were measured using a BCA method according to the manufacturer's instruction. Total TGF-β1 was quantified using a TGF-β1 ELISA kit (Invitrogen). Values were expressed as pg/mg protein for the protein extracts.

### Measurement of active serum 1, 25(OH)_2_ vitamin D_3_ in mice

Six-week-old male mice were anesthetized with ketamine and xylazine; blood was collected from the inferior vena cava, and the serum was separated by centrifugation at 7,500 g for 10 min at 4°C (n = 4). The serum level of 1, 25(OH)_2_ vitamin D_3_ was measured by RIA (SRL Inc., Tokyo, Japan).

### Cell culture

Primary proximal tubular epithelial cells were generated from the kidneys of WT, HT, and *kl/kl* mice using a method described previously^33^ with minor modification. Briefly, kidney cortices (6 wk male mice) were dissected, sliced, minced, and digested in 300 U/ml type II collagenase (Worthington Bio Corp) in FBS free DMEM in a shaking incubator at 37°C for 30 min. Collagenase was neutralized with 10% FBS/DMEM containing 100 U/ml penicillin and 0.1 mg/ml streptomycin. The suspension was triturated by pipetting and was passed through a 70 μm cell strainer (Becton Dickinson Labware, Franklin Lakes, NJ). The samples were centrifuged (500 rpm, 5 minutes) to pellet the tubules, washed with 10 ml FBS free-DMEM, centrifuged, and washed twice more. The final pellet, consisting mostly of renal tubules, was resuspended in culture medium (REBM bullet kit, Clonetics) and incubated at 37°C in a 5% CO_2_ incubator with medium changes every 2 days until approximately 80% confluent.

NRK52E cells were obtained from ATCC and cultured in DMEM with 5% FBS (Gibco) without antibiotic supplementation.

For TGF-β1 stimulation, cells underwent 24 h serum starvation and then were exposed to 5 ng/ml TGF-β1 (Cell signaling) for 12 or 24 h in the presence or absence of 0.2 or 2 μg/ml murine FGF23 (Peprotech) or 0.1 or1 μg/ml Calcitriol (Cayman).

To make high phosphate growth medium, an equal volume of 100 mM NaH_2_PO_4_ and 100 mM Na_2_HPO_4_ were mixed to make 100 mM Pi stocks; 2 mM and 4 mM Pi medium were made by dilution of Pi stocks with DMEM (Pi concentration in DMEM is approximately 1 mM). NRK52E cells were grown in normal DMEM with 5% FBS or high Pi DMEM with 5% FBS until 80% confluent. TGF-β1 stimulation was performed with serum free normal DMEM or high Pi DMEM.

### Quantitative Analysis

To quantify activated Smad3, the positive stained nuclei were counted in 20 consecutive high power fields (400×) and divided by the total number of nuclei. The number of collagen, and αSMA positive cells in the cortical interstitium was performed as described^36^. Band intensity in the western blots was measured using Image J software.

### Statistical Analysis

The data were analyzed with Student's *t*-test and one-way ANOVA with a Student–Newman–Keuls test (SPSS, 13.0) and expressed as means ± SD. For the data without Gaussian distribution, Kruskal-Wallis with post-hoc Mann-Whitney was used. P < 0.05 was considered to be statistically significant.

## Supplementary Material

Supplementary Information Supplementary information
