# Supplementary material for: Elevated serum 1,25(OH)2-vitamin D3 level attenuates renal tubulointerstitial fibrosis induced by unilateral ureteral obstruction in kl/kl mice
Source: Sci Rep. 2014 Oct 9;4:6563. doi: 10.1038/srep06563 (PMC5377451; doi:10.1038/srep06563)

Elevated serum 1,25(OH)$_2$-vitamin D$_3$ level attenuates renal tubulointerstitial fibrosis induced by unilateral ureteral obstruction in *kl/kl* mice

Yujing Sun[1, 2], Gengyin Zhou[2], Ting Gui[1], Aiko Shimokado[1], Masako Nakanishi[1], Kosuke Oikawa[1], Fuyuki Sato[1], and Yasuteru Muragaki[1*]

1First Department of Pathology, Wakayama Medical University School of Medicine, 811-1 Kimiidera, Wakayama 641-0011, Japan, 2Department of Pathology, School of Medicine, Shandong University, Jinan Wen Hua Xi Road 44, Jinan 250012, PR China.

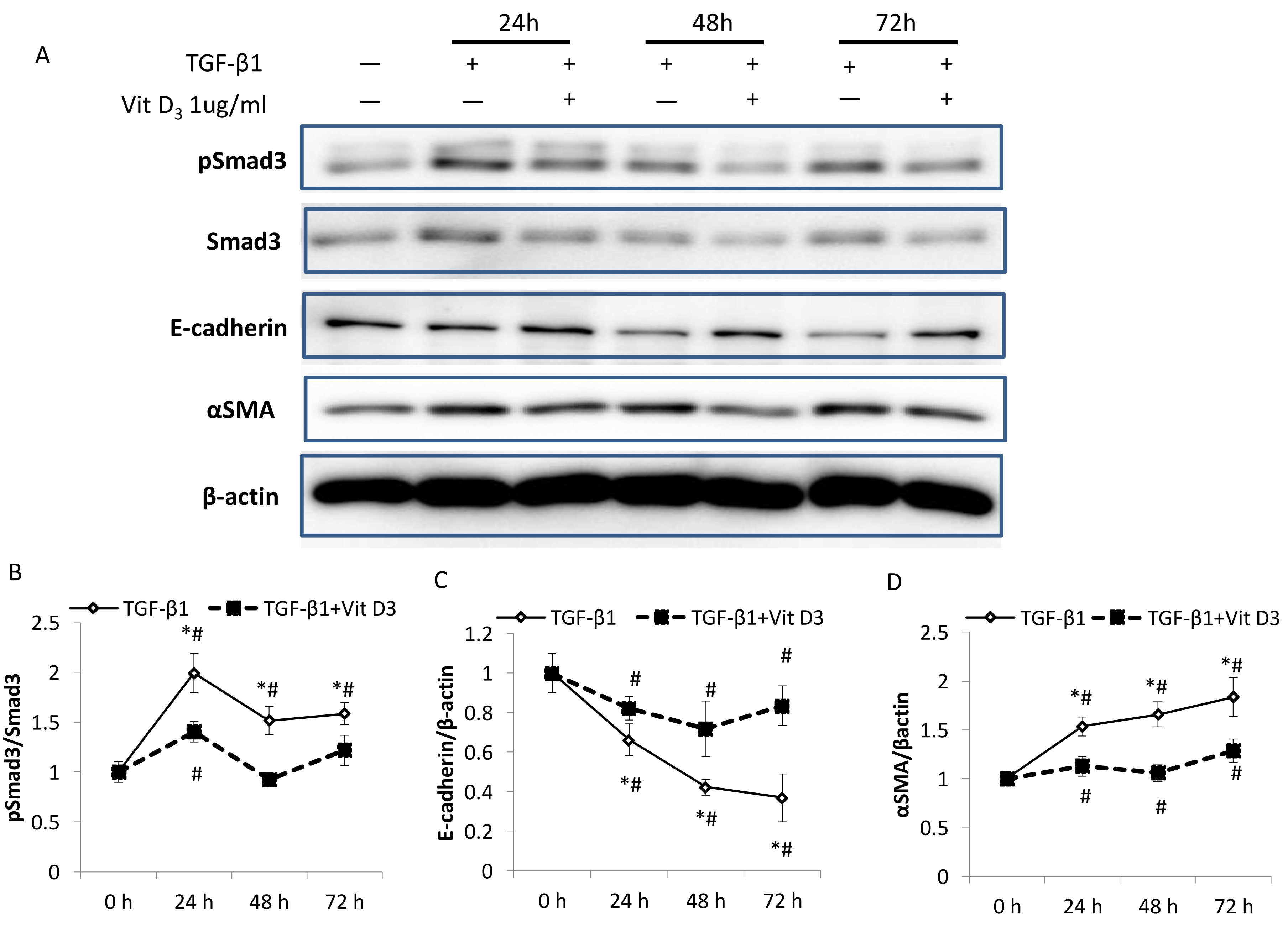

Supplementary figure 1

Supplementary figure 1. Long-term effects of 1,25 $(OH)_2$vitamin D_3 on TGF-β-induced EMT in NRK52E cells. NRK52E cells were treated with TGF-β1 (5ng/ml) for 3 days in the presence or absence of 1,25 $(OH)_2$vitamin D_3 (1ug/ml). (A) Representative Western blots for pSmad3, Smad3, E-cadherin, αSMA, and β-actin. (B-D) Summary of Western blot data for pSmad3 (B), E-cadherin (C), and α-SMA(D). Data are the means ± SD. *p<0.05 vs vitamin D_3 treated cells. #p<0.05 vs. control group. Experiments were repeated in triplicate.

Original whole panel for each western blot

Fig. 2C

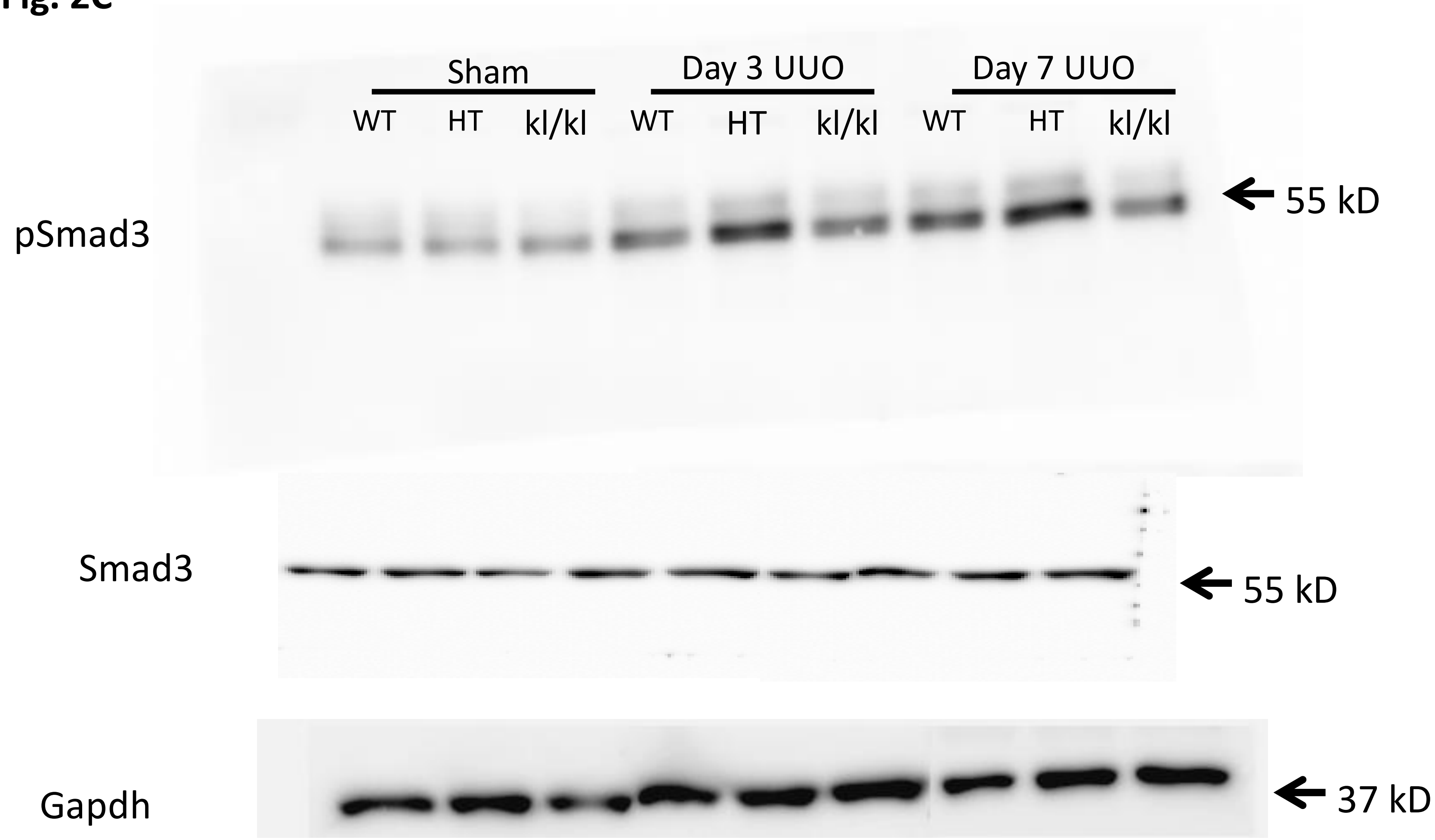

Original whole panel for each western blot

Fig. 3A

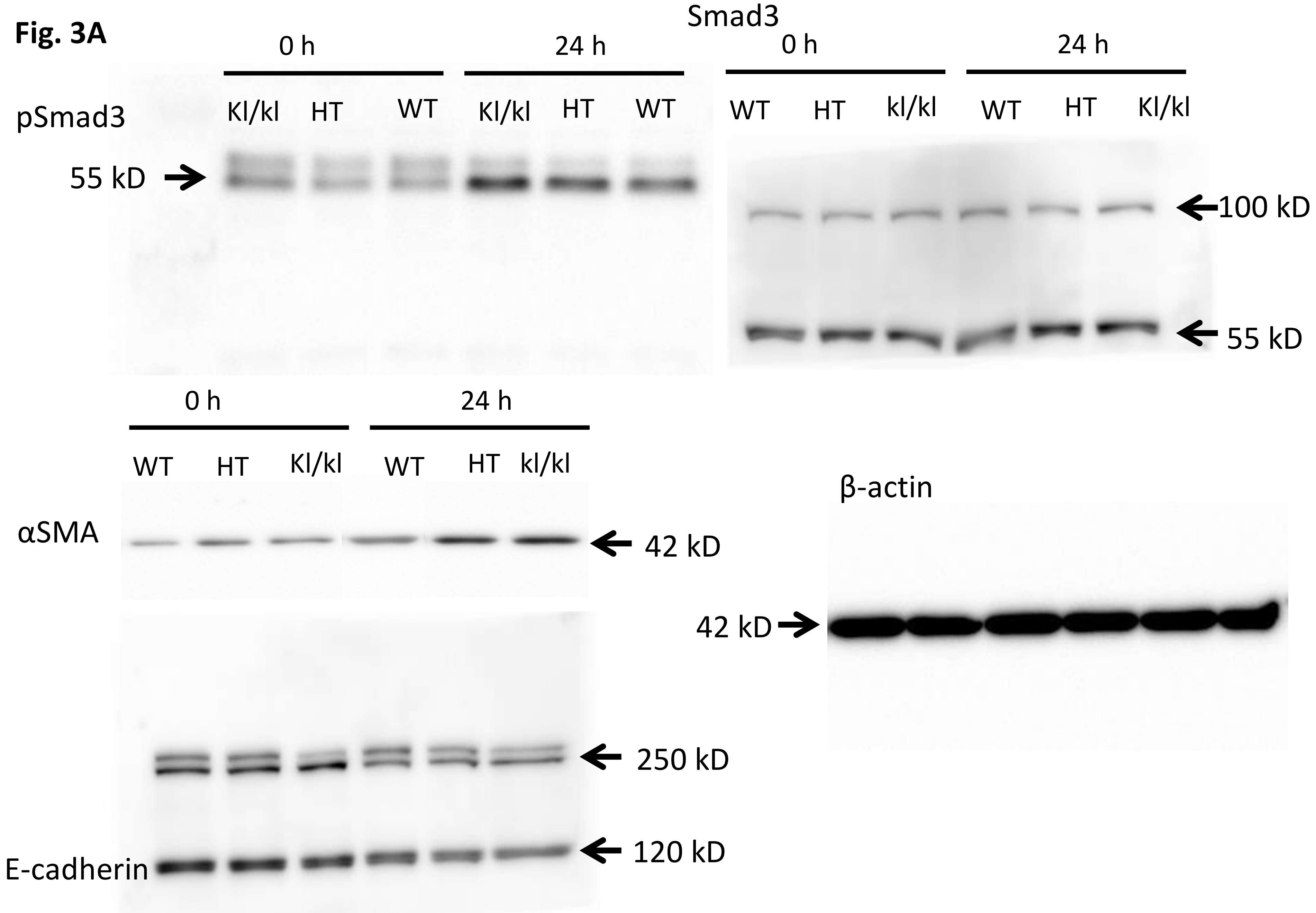

Original whole panel for each western blot

Fig. 4A

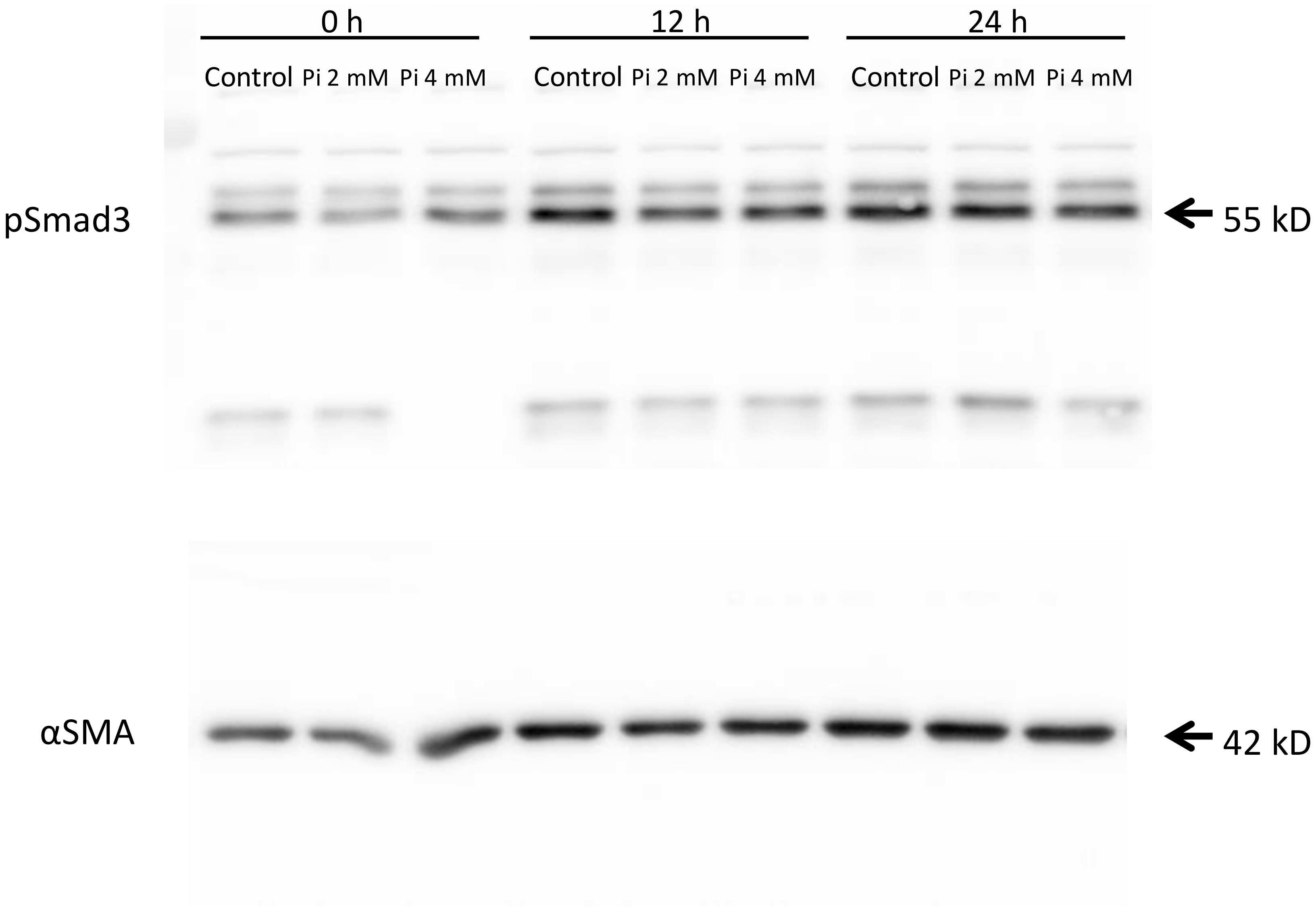

Original whole panel for each western blot

Fig. 4A

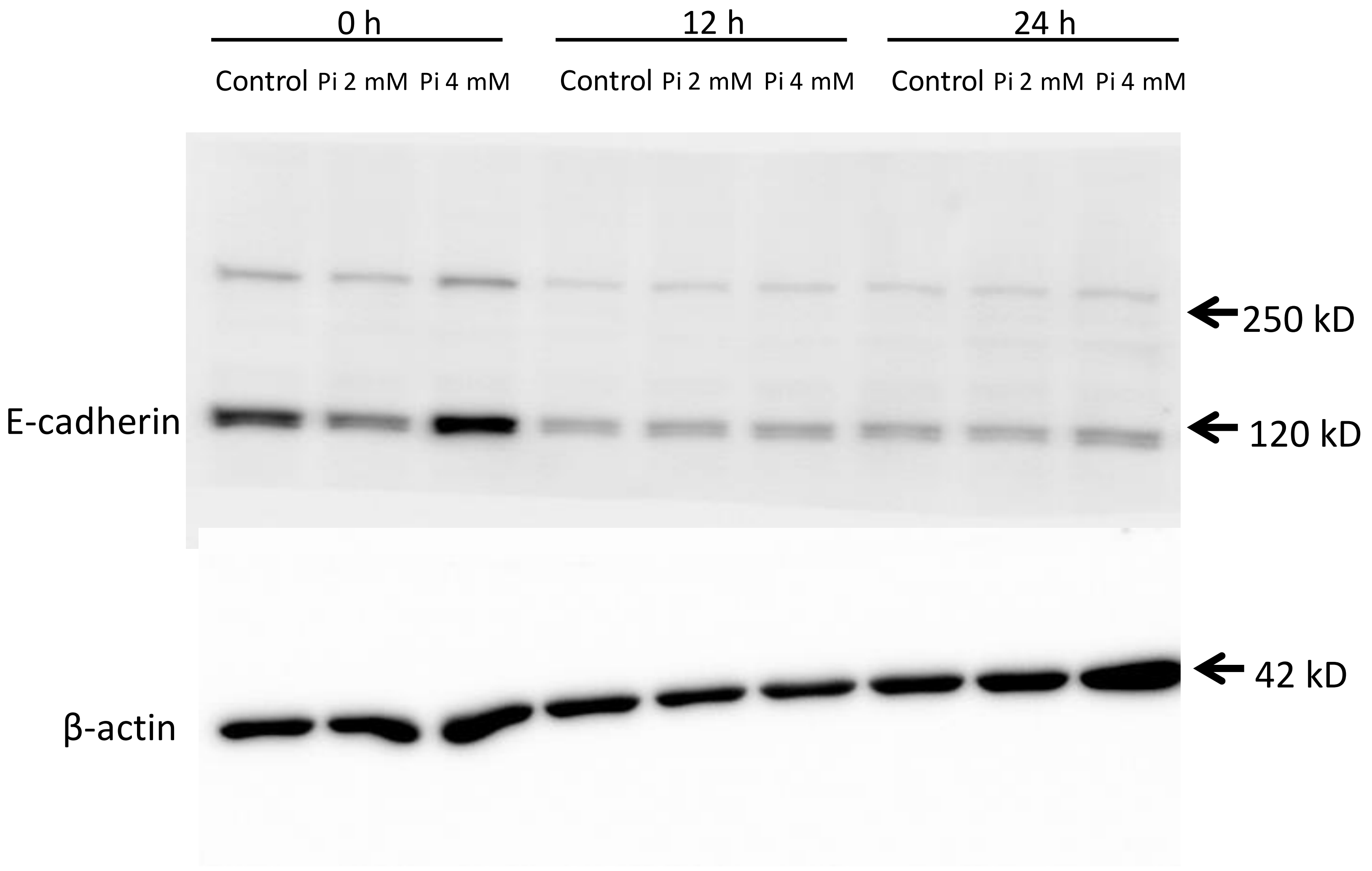

Original whole panel for each western blot

Fig. 4A

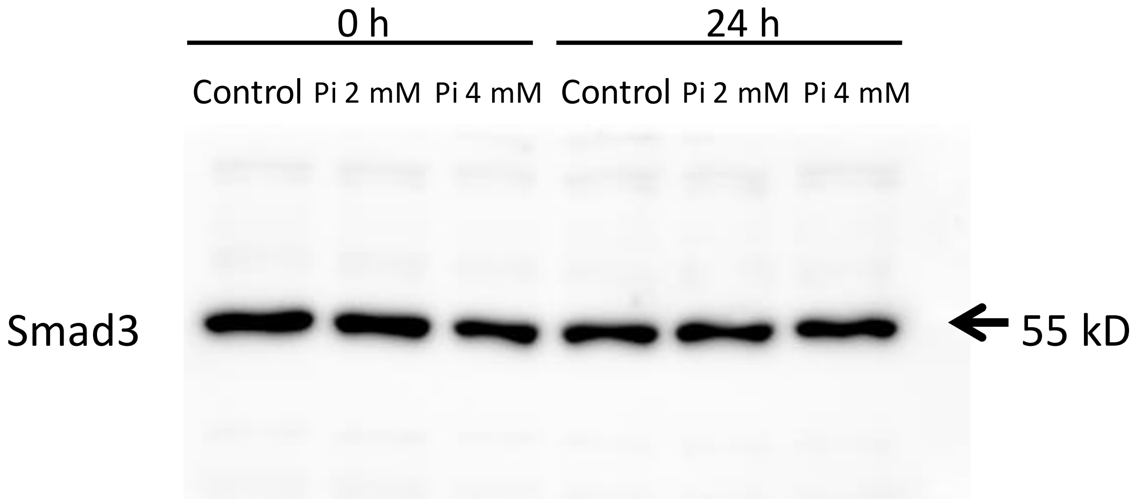

Original whole panel for each western blot

Fig. 5A

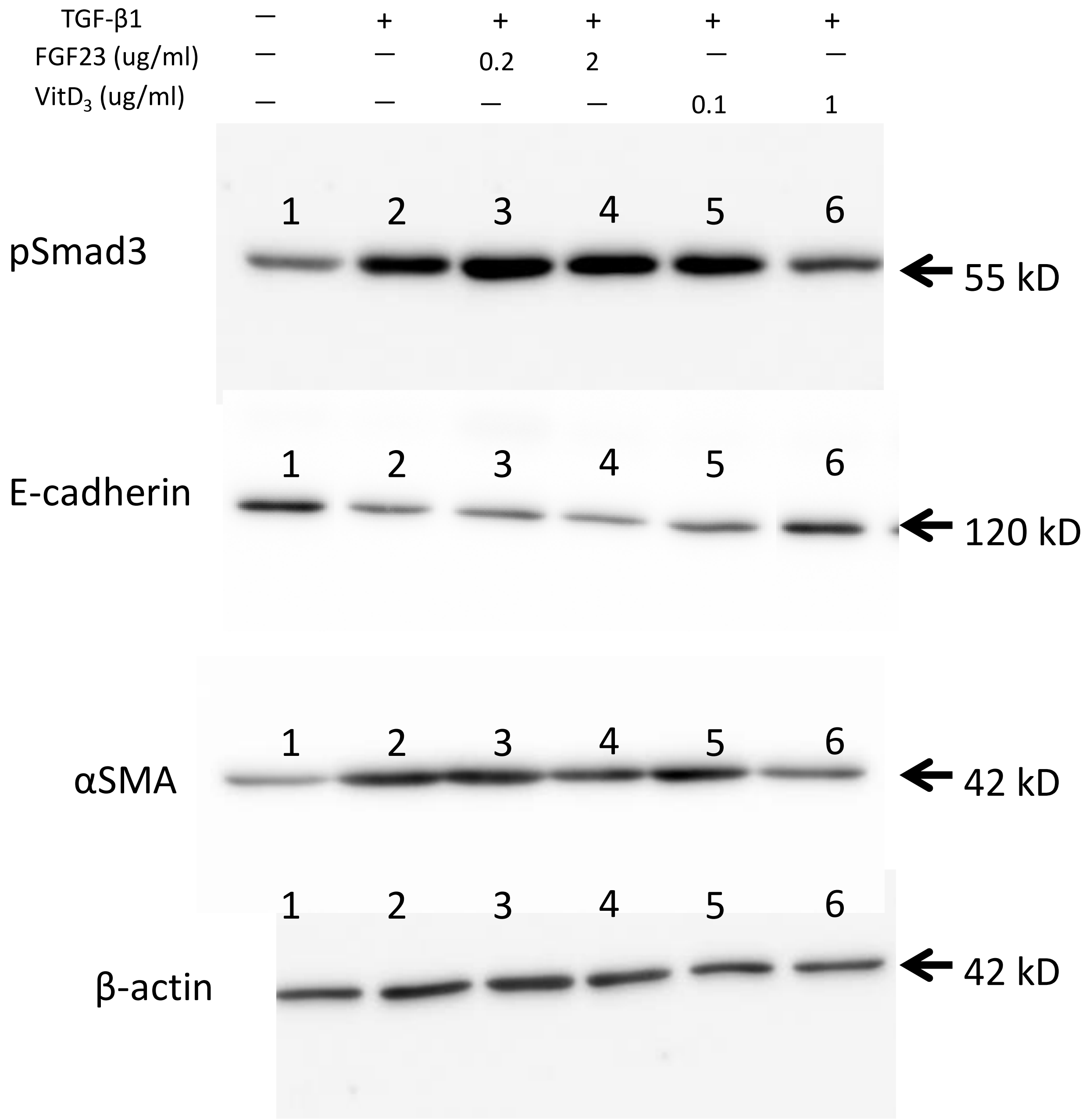

Original whole panel for each western blot

Fig. 5A

Smad3

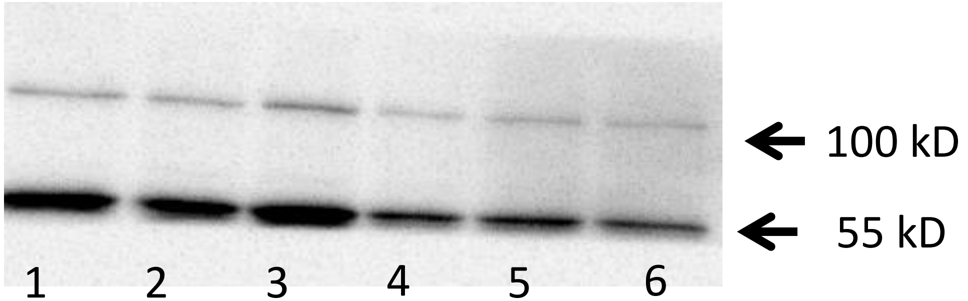

	1	2	3	4	5	6
TGF-β1	—	+	+	+	+	+
FGF23 (ug/ml)	—	—	0.2	2	—	—
$VitD_3$ (ug/ml)	—	—	—	—	0.1	1

Original whole panel for each western blot

Fig. 8B

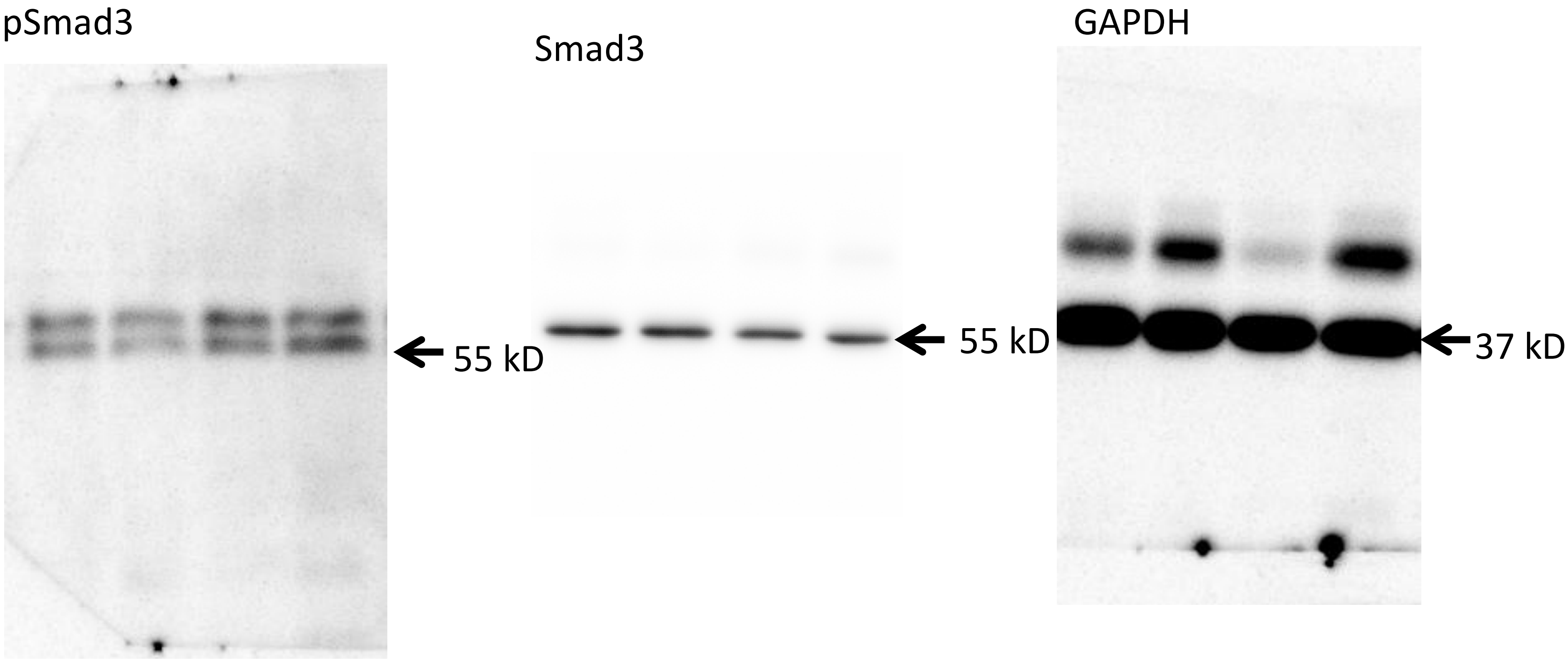

Two bands appeared in pSmad3 blotting. We have verified the upper band to be the non-specific band using tissues from Smad3-/- mice.

Original whole panel for each western blot

Supplementary figure 1 A

pSmad3

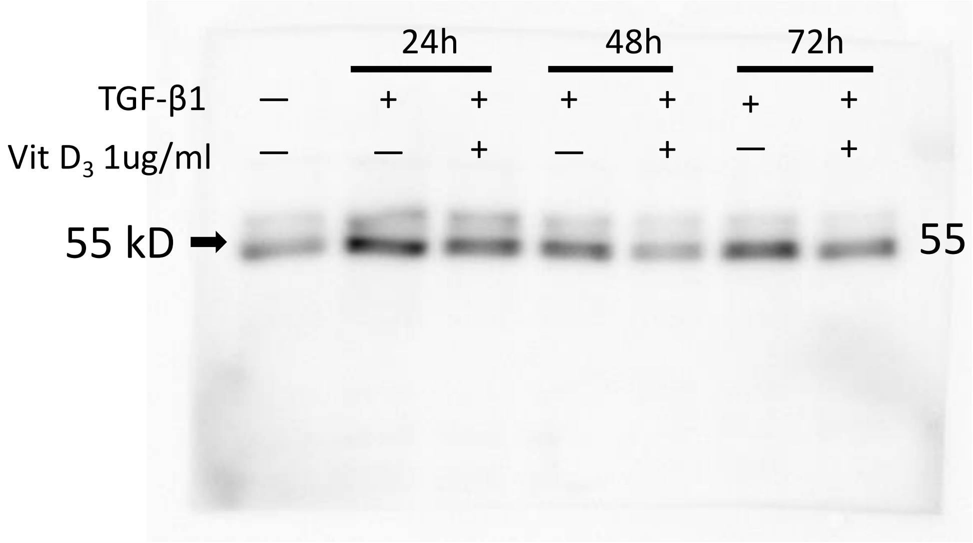

Smad3

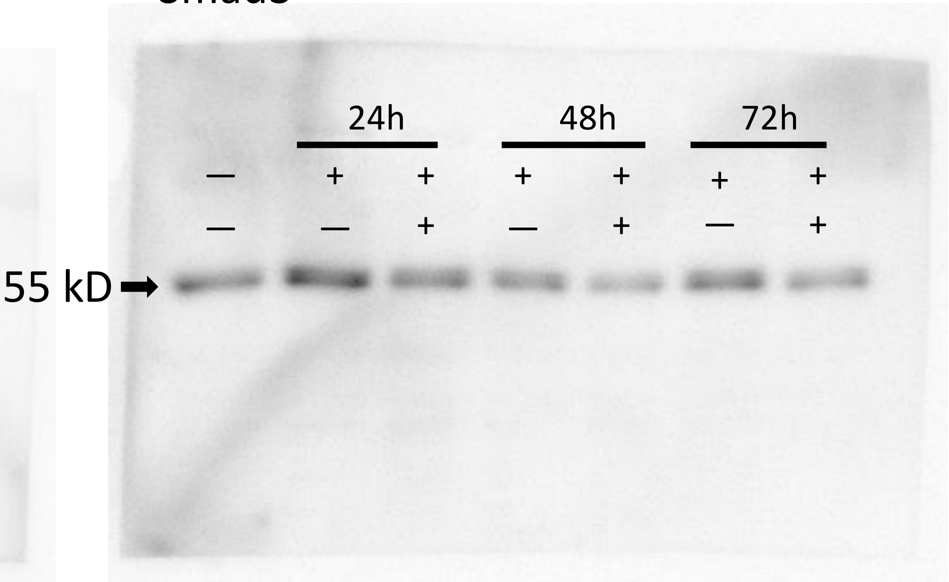

E-cadherin

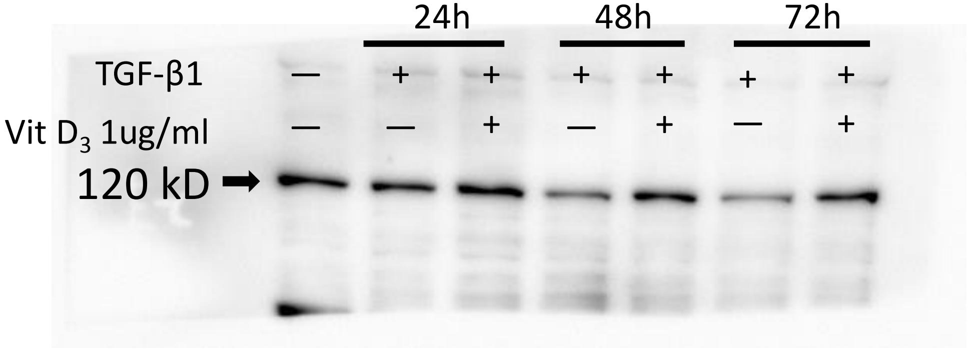

Original whole panel for each western blot

Supplementary figure 1 A

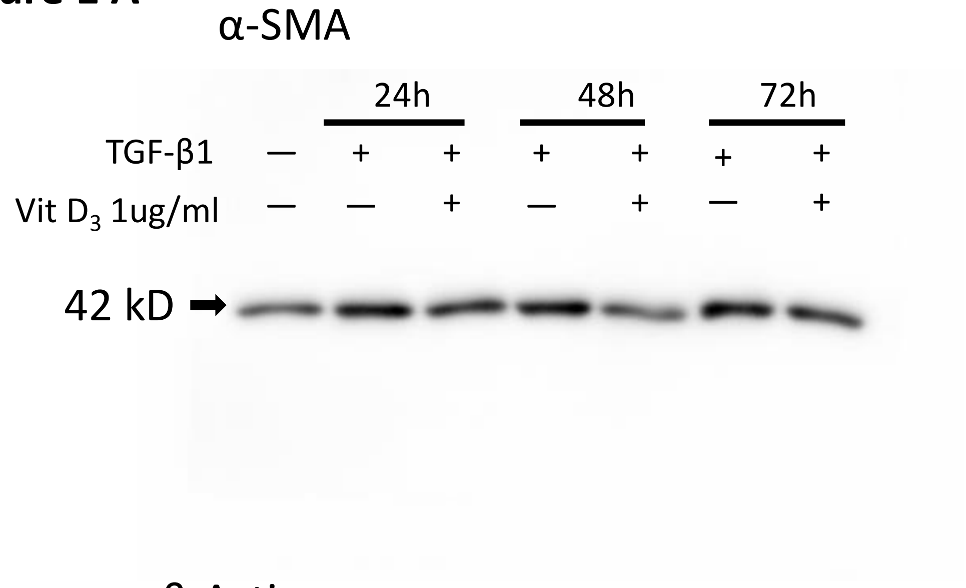

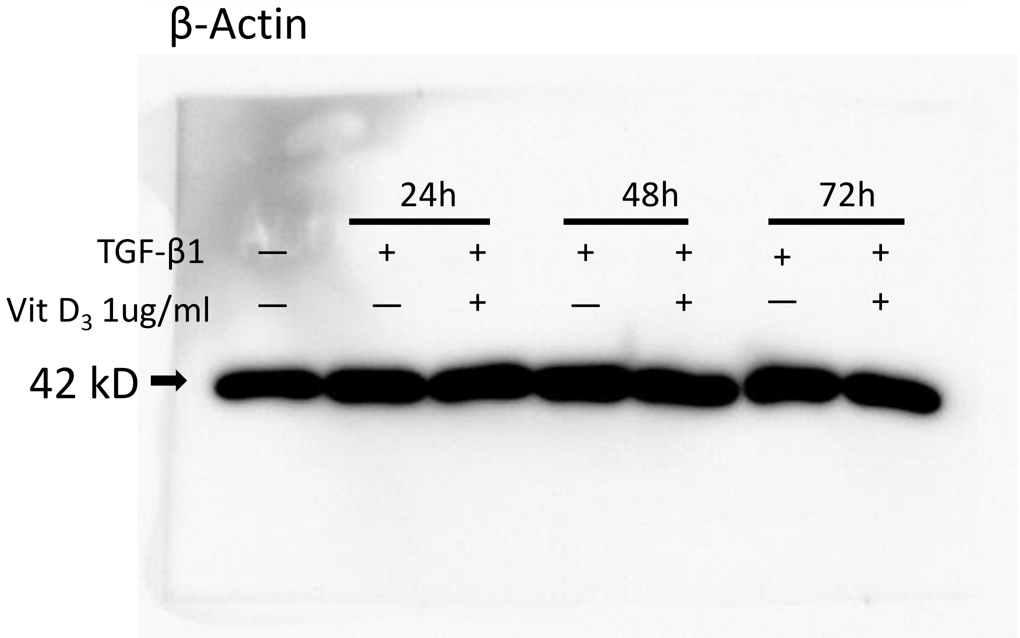

Supplement: Supplementary Information [file srep06563-s1.pdf]
